# Jorge Lobo’s disease with malignant degeneration to squamous cell carcinoma: case report^☆☆^

**DOI:** 10.1016/j.abd.2021.08.001

**Published:** 2021-11-23

**Authors:** Arival Cardoso de Brito, Maraya de Jesus Semblano Bittencourt, Thainá da Silva Gonçalves, Renata Henriques Cavalcante

**Affiliations:** a Service of Dermatology, Department of Dermatopathology, Universidade Federal do Pará, Belém, PA, Brazil; b Service of Dermatology, Universidade Federal do Pará, Belém, PA, Brazil

**Keywords:** Carcinoma, squamous cell, Lacazia, Lobomycosis

## Abstract

Jorge Lobo’s disease (JLD) is a chronic, granulomatous fungal infection caused by the traumatic implantation of the fungus *Lacazia loboi* in the cutaneous and subcutaneous tissues, with the presence of isolated nodular and coalescent keloidal lesions. Malignant degeneration is rare. This case report describes a 64-year-old male patient with JLD for 30-years who showed a change in the aspect of a lesion in the left lower limb. Histopathological examination confirmed the progression to well-differentiated squamous cell carcinoma (SSC). JLD is highly prevalent in tropical and subtropical regions, requiring monitoring concerning the transformation into SSC in long-term lesions.

Jorge Lobo’s disease (JLD) is a chronic granulomatous infection of the skin and subcutaneous tissue, without visceral dissemination, which is caused by the fungus *Lacazia loboi*.^1^, ^2^ This case report describes a 64-year-old male patient, agricultural worker, with JLD for 30 years, who had tumors with a keloidal appearance of different sizes, distributed throughout the skin, associated with isolated verrucous plaques (Figure 1, Figure 2). He had been undergoing systemic treatment with itraconazole for four years, with no improvement in the lesions. Six months ago, he noticed an alteration regarding the aspect of a lesion on the left leg, which, on dermatological examination, revealed an extensive vegetating, ulcerated plaque, with bleeding due to minor traumas (Fig. 3). Excision of the lesion and histopathological examination showed a well-differentiated, keratinizing, and ulcerated squamous cell carcinoma (SCC), adjacent to the acute and chronic inflammatory process with giant cells and abundant fungal yeast structures compatible with *Lacazia loboi* (Fig. 4). Left inguinal lymphadenectomy was also performed and the histopathological examination was compatible with metastatic SCC in one of the 13 examined lymph nodes, with capsular extravasation. Blood count was normal and serologies for hepatitis B and C and HIV viruses were negative; computed tomography of the chest and abdomen showed no alterations. He is currently being monitored at the oncology unit of a referral hospital.

**Figure 1:**
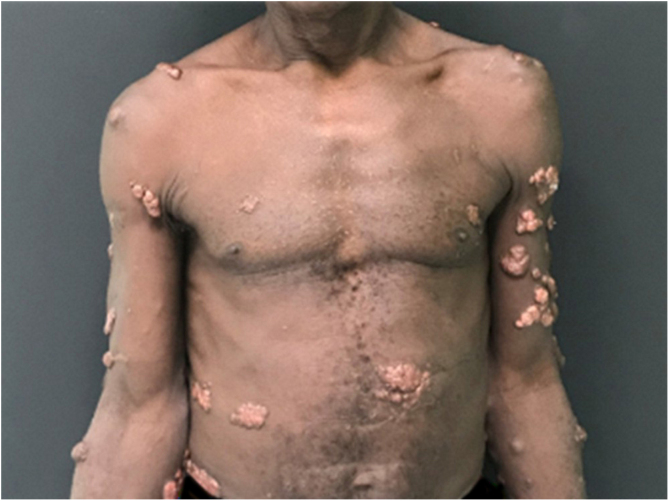
Multiple keloidal nodules disseminated on the skin.

**Figure 2:**
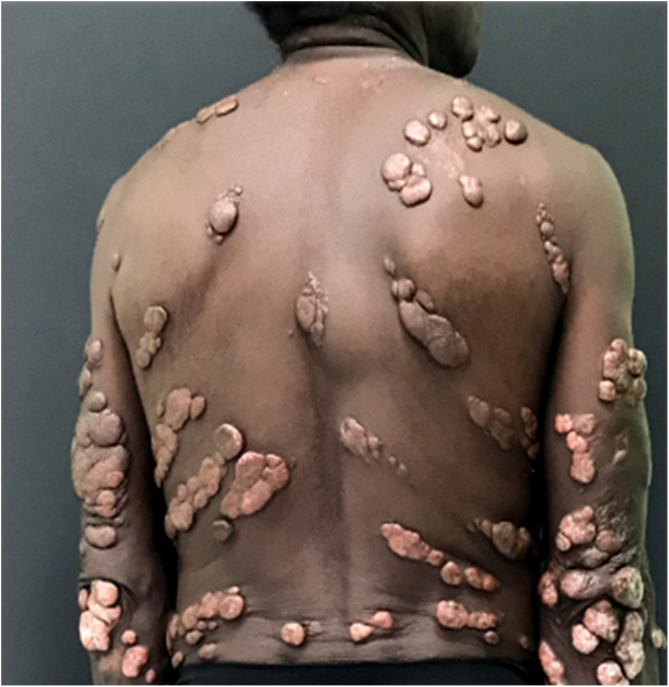
Multiple keloidal nodules and verrucous plaques disseminated on the skin.

**Figure 3:**
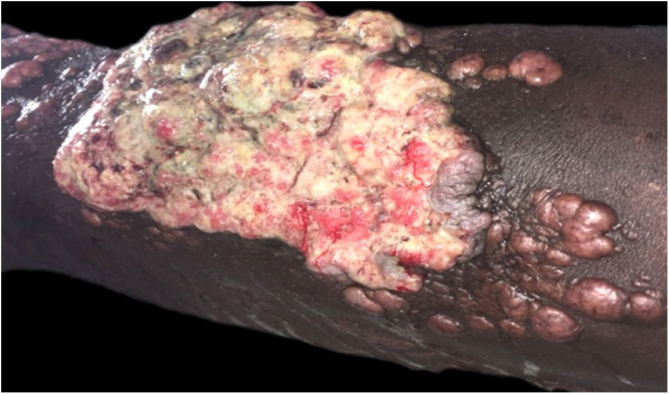
Vegetating, ulcerated, friable plaque located on the anterior portion of the left lower limb, measuring about 12 cm, with the presence of satellite keloidal lesions.

**Figure 4:**
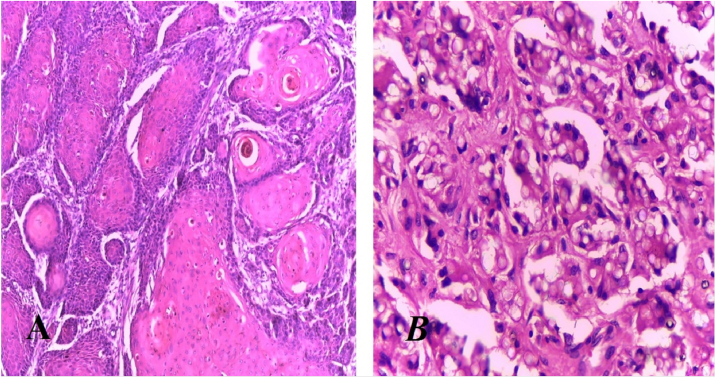
(A), Anatomopathological exam compatible with well-differentiated squamous cell carcinoma (Hematoxylin & eosin, ×40). (B), Dermis adjacent to the neoplastic lesion showing the presence of numerous fungal structures compatible with *Lacazia loboi* (Hematoxylin & eosin, ×100).

JLD, first described in 1931 by Jorge Lobo in a patient from the Amazon region, is a typical infection in tropical and subtropical areas, more common in men aged 20 to 40 years.^1^, ^2^ People who work in endemic areas and in contact with dense vegetation, high humidity, and high-temperature forests are susceptible to the disease. Clinically, it presents with polymorphic skin lesions, single or multiple, keloid-like nodules or plaques, which are the most frequent clinical forms. The lesions can progress with ulceration and secondary infection. There is a possibility of lymphatic and hematogenous spread, leading to multiple lesions.^2^ Old scars and chronic ulcers of any kind can lead to the development of SCC. The degeneration into SCC in JLD has been described in a few cases in the world literature and can occur after a long period of disease evolution.^2^, ^3^, ^4^, ^5^ One study showed two affected Brazilian natives from the Caiabi tribe who developed metastases and died, even after surgical resection.^3^ In the state of Pará, there have been reports in the literature of three patients with this transformation.^6^ The therapeutic approach to JLD remains challenging to this day. The therapy of choice depends on the clinical manifestation, ranging from systemic antifungals, electrocoagulation, cryotherapy to surgical excision with wide margins. Recurrence is frequent with any method. The use of triazoles such as posaconazole seems to be promising.^1^, ^2^, ^6^ Overall, the prognosis is good, but with aesthetic and/or functional impairment. The emergence of SCC in chronic lesions of scars and ulcers of different etiologies has been reported by many authors in patients with chronic granulomatous diseases, among which JLD should be included, and these patients should be submitted to closer surveillance of ulcerated and chronic lesions.^3^, ^4^, ^5^

## Financial support

None declared.

## Authors’ contributions

Arival Cardoso de Brito: Approval of the final version of the manuscript; design and planning of the study; effective participation in research orientation; intellectual participation in the propaedeutic and/or therapeutic conduct of the studied cases; critical review of the literature; critical review of the manuscript.

Maraya de Jesus Semblano Bittencourt: Approval of the final version of the manuscript; design and planning of the study; drafting and editing of the manuscript; collection, analysis, and interpretation of data; effective participation in research orientation; critical review of the manuscript.

Thainá da Silva Gonçalves: Approval of the final version of the manuscript; design and planning of the study; drafting and editing of the manuscript; collection, analysis, and interpretation of data; effective participation in research orientation; intellectual participation in the propaedeutic and/or therapeutic conduct of the studied cases; critical review of the literature.

Renata Henriques Cavalcante: design and planning of the study; drafting and editing of the manuscript; collection, analysis, and interpretation of data; effective participation in research orientation; intellectual participation in the propaedeutic and/or therapeutic conduct of the studied cases; critical review of the literature.

## Conflicts of interest

None declared.
